# Multidrug Efflux Pumps in Staphylococcus aureus: an Update

**DOI:** 10.2174/1874285801307010059

**Published:** 2013-03-22

**Authors:** Sofia Santos Costa, Miguel Viveiros, Leonard Amaral, Isabel Couto

**Affiliations:** 1 1Grupo de Micobactérias, Unidade de Microbiologia Médica, Instituto de Higiene e Medicina Tropical, Universidade Nova de Lisboa (IHMT, UNL), Portugal; 2 2Centro de Recursos Microbiológicos (CREM), UNL, Portugal; 3 3COST ACTION BM0701 (ATENS), Brussels, Belgium

**Keywords:** Antibiotics, biocides, efflux pumps, multidrug resistance, Staphylococcus aureus.

## Abstract

The emergence of infections caused by multi- or pan-resistant bacteria in the hospital or in the community settings is an increasing health concern. Albeit there is no single resistance mechanism behind multiresistance, multidrug efflux pumps, proteins that cells use to detoxify from noxious compounds, seem to play a key role in the emergence of these multidrug resistant (MDR) bacteria. During the last decades, experimental data has established their contribution to low level resistance to antimicrobials in bacteria and their potential role in the appearance of MDR phenotypes, by the extrusion of multiple, unrelated compounds. Recent studies suggest that efflux pumps may be used by the cell as a first-line defense mechanism, avoiding the drug to reach lethal concentrations, until a stable, more efficient alteration occurs, that allows survival in the presence of that agent.

In this paper we review the current knowledge on MDR efflux pumps and their intricate regulatory network in *Staphylococcus aureus*, a major pathogen, responsible from mild to life-threatening infections. Particular emphasis will be given to the potential role that *S. aureus* MDR efflux pumps, either chromosomal or plasmid-encoded, have on resistance towards different antimicrobial agents and on the selection of drug - resistant strains. We will also discuss the many questions that still remain on the role of each specific efflux pump and the need to establish appropriate methodological approaches to address all these questions.

## 1. INTRODUCTION

The introduction of antibiotics for the treatment of infectious diseases was one of the hallmarks in the 20^th^ century medicine. However, shortly after their introduction into the clinical practice, the first bacteria showing antibiotic resistance were described. Since then, the development of new antibiotics has been accompanied by the steady increase of antibiotic-resistant bacterial strains and the diversity of mechanisms used by bacteria to surpass the lethal effect of these compounds. Nowadays, at least one mechanism of resistance is described for each class of commonly used antibiotics [1]. Moreover, many bacterial species show multi- or pan-resistant phenotypes. Many of these multidrug resistant (MDR) bacteria can cause life-threatening infections, being a major concern both in the hospital and the community [2, 3]. *Staphylococcus aureus*, a Gram-positive cocci is one of the major bacterial pathogens, causing mild to life-threatening infections [4]. Besides its potential virulence, *S. aureus* also shows a remarkable diversity of resistance mechanisms towards antimicrobial agents [5]. Of major concern are the methicillin-resistant *S. aureus* (MRSA) strains, resistant to all beta-lactam antibiotics, which for many years caused major outbreaks in nosocomial environments and that are now increasingly isolated from the community, where they may also cause severe, many times fatal infections [6].

Bacteria can use different mechanisms of resistance to antibiotics, which include degradation or modification of the antibiotic; alteration of the bacterial target of the antibiotic; target protection and reduction of the intracellular concentration of the antibiotic, either by a decreased permeability of the cell wall or by the efflux of the antibiotic from the cell. Efflux-mediated resistance has been overshadowed in contrast with the other mechanisms known. However, it has been gathering more interest, as we recognize that many bacterial efflux pumps are able to extrude several, unrelated classes of antimicrobial compounds from the cell, promoting the appearance of multidrug resistance phenotypes [7, 8]. 

The physiological role of efflux pumps in bacteria has been related to the elimination of endogenous metabolites that are noxious to the cell, the secretion of virulence determinants, and in cell stress responses, suggesting that the drugs are “accidental substrates” of these transporters [9, 10].

Bacterial efflux systems can be either specific, extruding only one antibiotic or class of antibiotics, or be capable of extruding several classes of antimicrobial compounds, being designated multidrug resistance efflux pumps. These MDR efflux systems are classified in five families according to their energy requirements and structure namely, the major facilitator superfamily (MFS); the small multidrug resistance (SMR) family; the multidrug and toxic compound extrusion (MATE) family; the resistance-nodulation-cell division (RND) superfamily; and the adenosine-triphosphate (ATP)-binding cassette (ABC) superfamily. The transporters of the first four families are secondary transporters that use the proton motive force to drive the extrusion of their substrates by an antiport H^+^:drug mechanism, with the exception of the MATE family, which can also use the sodium membrane gradient as the source of energy. On the other hand, the transporters of the ABC superfamily are primary transporters that use ATP to drive the extrusion of their substrates [11].

To date, more than ten multidrug efflux pumps have been described for *S. aureus*, encoded either in the chromosome or in plasmids (Table **1**). This review summarizes the most recent data on *S. aureus* multidrug efflux pumps, focusing on their contribution to antimicrobial resistance of *S. aureus* strains. 

## 2. CHROMOSOMALLY-ENCODED MDR EFFLUX PUMPS

### 2.1. NorA

The multidrug efflux pump NorA is one of the most studied efflux systems in *S. aureus*. The chromosomal gene that codes for it, *norA*, was first described in a fluoroquinolone-resistant isolate collected in 1986 at a Japanese hospital [12]. The *norA* gene presents some genetic diversity, with three *norA* alleles described to date that differ up to 10% in the nucleotide sequence [13-15]. NorA is a 388 aminoacid protein that comprises 12 transmembrane segments, belongs to the MFS and shares 44% identity with the multidrug efflux pump Bmr from *Bacillus subtilis* and 24% identity with the tetracycline efflux pump Tet(A) from *Escherichia coli* [16,17]. Several studies have shown that NorA can extrude an array of chemically and structurally dissimilar compounds, namely hydrophilic fluoroquinolones, such as norfloxacin and ciprofloxacin, dyes, like ethidium bromide and biocides, such as quaternary ammonium compounds [16,18,19]. It is known that *norA* has a basal level of expression, which accounts for some level of reduced susceptibility towards these antimicrobial compounds [19,20]. Increased resistance to fluoroquinolones, biocides and dyes has been associated with NorA-mediated efflux, *via* the increased expression of the *norA* gene [19,21,22]. This increased expression can be either constitutive, through the acquisition of mutations in the *norA* promoter region, or inducible, through the action of regulatory proteins [20,23,24]. The first mutations to be described on *norA* promoter region were punctual mutations occurring 89 bp upstream of the initiation codon and downstream of the -10 motif, in the 5’-UTR region [19,21]. It has been suggested that these mutations may lead to an increase of the *norA* mRNA half-life, through an alteration of the mRNA secondary structure, which could be less sensible to the action of RNases [25]. Other studies with strains carrying these punctual mutations have detected, instead, an increase in *norA* transcripts resulting from an increased rate of *norA* transcription [26]. More recently, several groups have reported the occurrence of deletions and insertions in this same region, which may lead to increased *norA* mRNA stability [22,27,28]. To date, no correlation was found between antimicrobial resistance and mutations occurring in *norA* coding region [13,15]. Besides alterations occurring in *norA* promoter region, the production of NorA may also be regulated by several regulatory systems, albeit a clear regulatory pathway remains to be elucidated (see Part 4).

Similarly to other efflux pumps belonging to the MFS, NorA uses the proton motive force to energize the transport of antimicrobial compounds across the cell membrane, *via* an H^+^:drug antiport mechanism. Studies have shown that the NorA-mediated efflux of ethidium bromide [18] and norfloxacin [19,21] is sensible to protonophores, such as carbonyl *m*-chlorophenylhydrazone (CCCP), which dissipates the membrane proton gradient. It was also demonstrated that the accumulation of norfloxacin in *E. coli* everted vesicles carrying a functional NorA was affected by the presence of nigericin, which also dissipates the membrane proton gradient, but not by valinomycin, which collapses the electric gradient, supporting the hypothesis that NorA-mediated efflux is coupled to the proton gradient across the membrane [21]. 

### 2.2. NorB

Since the first studies in NorA, evidence was gathered for the occurrence of other efflux systems in the chromosome of *S. aureus* [29,30]. The efflux pump NorB is structurally similar to the efflux pumps Blt (41%) and Bmr (30%) from *B. subtilis*, as well as to the *S. aureus* NorA (30%) and QacA (39%). It is a MFS proton-driven efflux pump composed by 463 aminoacids, with 12 transmembrane segments. NorB confers resistance to some of the NorA substrates, such as hydrophilic fluoroquinolones (norfloxacin and ciprofloxacin), biocides (tetraphenylphosphonium and cetrimide) and the dye ethidium bromide, as well as to non-NorA substrates, such as the hydrophobic fluoroquinolones moxifloxacin and sparfloxacin, and to tetracycline [31]. A study with a mouse subcutaneous abscess model showed that NorB was important for *S. aureus* fitness, suggesting a putative role for NorB in staphylococcal pathogenesis [32]. Following studies from the same group propose that NorB may be involved in *S. aureus* response to acid shock and reduced aeration, conditions which triggered overexpression of *norB* gene [33,34]. In both cases, the overexpression of *norB* was associated with an increased resistance to NorB substrates, such as moxifloxacin [33,34]. In parallel with NorA, the regulation of NorB involves diverse regulatory pathways (see Part 4).

### 2.3. NorC

The efflux pump NorC is codified by the chromosomal gene *norC*. It is a 462 aminoacid protein with 12 transmembrane segments that belongs to the MFS and shares 61% identity with NorB [35]. NorC is associated with low-level resistance towards hydrophilic and hydrophobic fluoroquinolones, such as ciprofloxacin, moxifloxacin and garenoxacin, and to the dye rhodamine [35,36]. Studies have indicated that the wild-type expression of *norC* is apparently not sufficient to affect the susceptibility towards these compounds, and that low-level resistance is achieved through *norC* overexpression [35]. 

### 2.4. MepA

The efflux pump MepA was identified in studies with *S. aureus norA* disrupted mutants [37]. MepA is encoded by the chromosomal gene *mepA* and it was the first multidrug transporter from the MATE family to be described in *S. aureus*. This 451 aminoacid protein has 12 transmembrane segments and presents 26% and 21% identity to the MATE transporters CdeA from *Clostridium difficile* and NorM from *Vibrio parahaemolyticus*, respectively. MepA was found to be associated with a MDR phenotype, conferring low-level resistance to quaternary ammonium compounds, such as benzalkonium chloride, cetrimide, dequalinium, tetraphenylphosphonium, pentamidine and the dye ethidium bromide, to chlorhexidine, pentamidine, as well as to tigecycline, an antibiotic from the class of the glycylcyclines. The fluoroquinolones ciprofloxacin and norfloxacin were shown to be weak substrates of MepA [37,38].

The *mepA* gene is integrated in the *mepRAB *operon*.* Sequence analysis revealed that the encoded protein MepR is similar to regulatory proteins from the MarR family [37]. No significant similarity was found between MepB and any other protein with known function and no association was found between MepB and MDR phenotypes [37].

### 2.5. MdeA

The *S. aureus *chromosomal gene *mdeA*, which encodes the efflux pump MdeA, was identified in an open reading frame expression library of the *S. aureus* genome [39]. MdeA has 479 aminoacids, possesses 14 transmembrane segments and belongs to the MFS, using the proton motive force to energize the transport of its substrates. MdeA shares 37% identity with the efflux pump LmrB from *B. subtilis*, 24% with EmrB from *E. coli* and 23% with QacA from *S. aureus* [39]. The overexpression of *mdeA* was associated to increased resistance to the biocides benzalkonium chloride, dequalinium and tetraphenylphosphonium, to the dye ethidium bromide, and to the antibiotics virginiamycin, novobiocin, mupirocin and fusidic acid [39]. A subsequent study showed that the fluoroquinolones norfloxacin and ciprofloxacin are weak substrates of this pump [40]. 

The overexpression of *mdeA* can be achieved by the occurrence of mutations in the *mdeA* promoter region, producing however only a slight increase in the MDR resistance phenotype [39].

### 2.6. Other Chromosomally-encoded MDR Efflux Pumps 

The protein SepA, codified by the chromosomal gene *sepA*, has been identified as an efflux pump that confers low-level resistance to antiseptic compounds, namely benzalkonium chloride, chlorhexidine gluconate and the dye acriflavine [41]. This transporter comprises 157 aminoacids and four putative transmembrane segments, a characteristic of the transporters from the SMR family. However, the conserved motifs of this family are not present in SepA, although some residues important for the transport specificity and for the antiport H^+^:drug are present in a different location, suggesting that SepA may belong to an yet identified family of transporters [41].

SdrM is an efflux pump codified by the chromosomal gene *sdrM*. SdrM shares 23% and 21% identity with the *S. aureus* MDR efflux pumps NorB and QacA, respectively. Sequence analysis postulate that SdrM may belong to the MFS, possessing 14 transmembrane segments. This efflux pump was shown to be associated with low-level resistance to acriflavine, ethidium bromide and to the fluoroquinolone norfloxacin by promoting an energy-dependent efflux of these compounds [42]. 

More recently, Floyd and colleagues described another *S. aureus* multidrug efflux pump, LmrS (lincomycin resistance protein of *S. aureus*), with 39% identity with the lincomycin resistance protein LmrB of *B. subtilis*, and 25% identity with the efflux pumps FarB of *Neisseria gonorrhoeae* and EmrB of *E. coli *[43]. LmrS was described as a 480 aminoacid MFS protein, with 14 predicted membrane-spanning domains, being involved in increased resistance to linezolid and tetraphenylphosphonium chloride, sodium dodecyl sulfate, trimethoprim, and chloramphenicol [43].

## 3. PLASMID-ENCODED MDR EFFLUX PUMPS

### 3.1. QacA/B

In the early 1980s, a gene encoding resistance to several antiseptics and disinfectants was identified on plasmid pSK1, carried by clinical isolates of *S. aureus* [44]. This gene, later designated *qacA*, encodes the efflux pump QacA that comprises 514 aminoacids and is a member of the MFS, presen-ting 14 transmembrane segments [45]. The *qacA* gene is found in large conjugative plasmids not only from *S. aureus* strains, but also from coagulase-negative staphylococci [46]. QacA mediates resistance to a wide array of antimicrobial compounds, more than 30 lipophilic, mono- and divalent cations, that belong to 12 distinct chemical classes, including dyes, such as ethidium bromide and rhodamine; quaternary ammonium compounds, like benzalkonium chloride, tetraphenylphosphonium and dequalinium; diamidines, such as pentamine and DAPI; biguanidines, like chlorhexidine; and guanylhydrazones [47-49]. The transport of these substrates is driven by the proton motive force *via* an antiport H^+^:drug mechanism [47,50]. 

Another antiseptic resistance determinant, the gene *qacB*, has been found in plasmids, isolated from strains of clinical origin dating from the 1950s [51]. The *qacB* gene encodes for the efflux pump QacB and was first described on plasmid pSK23 [52]. *qacB* is closely related to the *qacA* gene, differing in only seven nucleotides in the entire sequence. Even so, the substrate specificity of both pumps varies, with QacB conferring resistance to only monovalent lipophilic cations [52,53]. Mutagenesis analysis revealed that the presence of aspartic acid at the position 323 of QacA, located within the transmembrane segment 10, instead of an alanine in QacB, is critical for conveying resistance to divalent cations. Moreover, it indicates that an acidic charge in that position is essential for the binding of divalent substrates and that QacA has two different binding sites for substrates [53,54], which was also confirmed by kinetic analysis of the QacA transport of monovalent and divalent fluorescent substrates [50].

Analysis of the *qacA/B* genes carried by *S. aureus* clinical isolates has revealed the occurrence of some genetic variability of these genes and that these variant forms of either QacA or QacB may confer different levels of resistance to biocides and dyes [55]. In addition, a recent study indicated that a clinical isolate harboring a variant of QacB, that carried a glutamic acid instead of alanine in position 320, which is located in the transmembrane segment 10, conferred low-level resistance to the hydrophilic fluoroquinolones norfloxacin and ciprofloxacin [56].

### 3.2. Smr

The efflux pump gene *smr* was identified in the late 1980s by different groups in several plasmids conferring antiseptic and ethidium bromide resistance. This gene was designated *ebr* [57], *qacC/D* [58] or *smr* [59], but sequence analysis of the determinants described by these authors revealed that they were all identical. This gene can be found in either small non-conjugative plasmids, such as pSK89, or large conjugative plasmids, like pSK41, in both *S. aureus* and coagulase-negative staphylocci, and encodes the efflux pump Smr [46,58,60]. 

Hydropathy analysis revealed that Smr, with 107 aminoacids, has four transmembrane segments and belongs to the SMR family that uses the proton motive force to energize the transport of noxious compounds [61]. This efflux pump conveys low-level resistance to a narrower number of compounds when compared with QacA/B, namely monovalent cationic dyes, such as ethidium bromide, and quaternary ammonium compounds, such as benzalkonium chloride [61].

Due to its small size, it was questioned if Smr, as a monomer, would be able to conduct the transport of the substrates. *In vitro* transport assays with purified Smr and site-directed mutagenesis, showed that it is capable of performing efflux, without however clarifying if as a monomer or as an oligomer [61,62], as described for other pumps of the SMR family [63]. 

Sequence analysis of the *smr* gene found in several plasmids showed that it is highly conserved, with only one variant, *smr’*, which shows a single nucleotide alteration that results in the change of an alanine by a serine at residue 9, producing the Smr’ protein. It was demonstrated that Smr’ conveyed a resistance phenotype similar to Smr [64]. However, the regions flanking *smr* showed some diversity, leading to the classification of *smr* cassette-like structures (comprising the *smr* gene flanked by direct repeats) in three distinct groups (types 1 to 3), apparently with no differences at the type or level of resistance conferred [60,65,66]. 

### 3.3. Other Plasmid-encoded MDR Efflux Pumps

Other *S. aureus* plasmid-borne efflux pumps conferring resistance to antiseptics and disinfectants include QacG, QacH and QacJ.

The efflux pump gene *qacG* was identified in *S. aureus* isolates collected in the food industry. This gene was located on the 2.3 kb plasmid pST94. It encodes the efflux pump QacG that has 107 aminoacids and four transmembrane segments, shares 69.2% identity with the efflux pump Smr and belongs to the SMR family of transporters [67]. The determinant *qacH* was first detected in a 2.4 kb plasmid isolated from a *Staphylococcus saprophyticus* strain from the food industry. The *qacH* gene shares 76% and 70% nucleotide identity to the *smr* and *qacG* genes, respectively, and encodes the efflux pump QacH, with 107 aminoacid residues and four transmembrane segments, that belongs to the SMR family [68]. The efflux pump gene *qacJ* was identified on a 2.65 kb plasmid found in several strains from three staphylococcal species; *S. aureus*, *Staphylococcus simulans* and *Staphylococcus intermedius*, all collected from horses [69]. The encoded efflux pump QacJ also belongs to the SMR family, with 107 aminoacids and four transmembrane segments, and shares 72.5%, 82.6% and 73.4% identity with the efflux pumps Smr, QacG and QacH, respectively.

Despite the differences found between the aminoacid sequences of Smr and QacG/H/J, all these pumps share almost identical substrate specificities, conferring similar levels of resistance to benzalkonium chloride, ethidium bromide and cetyltrymethylammonium bromide [69].

## 4. REGULATION OF *S. aureus* MDR EFFLUX PUMPS

### 4.1. Global Regulators

The regulation of *S. aureus* MDR efflux pump genes is complex and is affected by several global regulators that act in an intricate regulatory network and are involved in chemical and physical stress response as well as in *S. aureus* pathogenesis.

The regulator MgrA, previously named NorR or Rat, was first identified by its binding to the *norA* promoter region [70]. MgrA is highly homologous to MarR family proteins and in a lesser-extent to SarA family proteins, both transcriptional regulatory proteins that possess a helix-turn-helix motif involved in the specific binding to DNA [71,72]. MgrA is a pleiotropic regulator that uses an oxidation-sensing mechanism [73] in the regulation of autolysis, virulence genes, antibiotic resistance genes (efflux pumps) and other genes involved in the *S. aureus* metabolism [74,75]. This regulator was also found to modulate the expression of other global regulators, such as SarA, which affects the expression of virulence determinants [76] and the alternative sigma factor SigB, essential for the *S. aureus* chemical and stress response [77,78]. These findings suggest that some modulation effects attributed to MgrA may be achieved in an indirect manner [75]. 

MgrA regulates three *S. aureus* MDR efflux pumps; NorA, NorB and NorC, as well as the non-MDR tetracycline efflux pump Tet38 and the ABC efflux pump AbcA [31,79,80]. Reports from different groups studying MgrA had reported divergent effects of this regulator on the *norA* and *norB* expression in genetic backgrounds that differed in the *rsbU* locus, that encodes the protein RsbU from the serine/threonine phosphatase family [26,31,70,75,79]. A thorough examination of these genetic systems evidenced that MgrA can be post-translationally phosphorylated by the putative serine/threonine kinase PknB [81], and that the phosphorylated MgrA-P can be dephosphorylated by RsbU [82], altering the ability of MgrA to bind to the *norA/B* promoters. A model for MgrA *norA/B* modulation was proposed after the demonstration that only MgrA was able to bind to the *norA* promoter and only MgrA-P could bind to *norB* promoter. According to this model, MgrA acts as a *norA* repressor, and upon phosphorylation, MgrA-P is released from the *norA* promoter, allowing *norA* transcription. The MgrA-P will, in turn, bind to the *norB* promoter, acting as a repressor of *norB* [82]. The cellular ratio between MgrA and MgrA-P (and hence their effect on efflux pump expression) is dependent of PknB and RsbU, which in turn are modulated by the alternative sigma factor SigB, involved in *S. aureus* stress response.

It was proposed that NorC may be negatively regulated by MgrA, acting in a concerted manner with NorB. However, no MgrA recognition sites were found in the *norC* promoter region, suggesting that MgrA may have an indirect effect over *norC* expression and that other factors may play a role in the production of this efflux pump [35].

The transcriptional regulator NorG, from the GntR-like family, was identified by binding to the *norA* promoter in a *mgrA*^-^ background [80]. It was shown that NorG was able to bind specifically to the promoters of the efflux pump genes *norA*, *norB*, *norC*,* abcA* as well as to its own promoter. No binding of NorG to the *tet38* or *mgrA* promoters was detected [80]. However, MgrA may bind to the *norG* promoter, thus affecting its expression [80]. Despite the binding of NorG to the promoter regions of *norA*, *norB*, *norC *and* abcA,* overexpression of *norG* was accompanied only by a mild increase in *norB* expression, suggesting that NorG is an activator of *norB* [80]. An analysis of the transcriptional profile of NorG confirmed its role as an activator of NorB and revealed that NorG affects negatively the expression of both *norC* and *abcA* [36]. It also showed that NorG activates the transcription of the global regulators *mgrA*, *arlS* and *sarZ*. SarZ, a MgrA homologue involved in the oxidative stress response of *S. aureus* was previously shown to downregulate *norB* and *tet38* expression [83]. It has been documented that NorG is absent from some prototype *S. aureus* strains, such as MW2, MSSA476 and MRSA 252, showing that NorG may not be essential for efflux pump regulation in these strains [84].

The expression of the NorA and NorB can be also modulated by the two-component regulatory system ArlRS, involved in adhesion, autolysis and extracellular proteolytic activity of *S. aureus* [70,85,86].

A recent study revealed that *norA* expression can be additionally modulated by *fur*, a ferric uptake regulator that has a putative binding site in the *norA* promoter region. It also showed that NorA is iron responsive and may contribute to the export of siderophores in *S. aureus* [87].

### 4.2. Specific Regulators

In addition to global regulators described above, the transcription of the genes coding for some *S. aureus* MDR efflux pumps can also be modulated by specific regulators. This is the case for MepA and QacA/B, regulated by MepR and QacR, respectively. Both these regulators function as sensors, binding to the substrates of the MDR efflux pumps and inducing their expression, thus acting as substrate-responsive regulators.

MepR is a self-repressive protein that binds to sequences in which pseudo palindromes are present, as well as to the motif GTTAG, both located in the promoter regions of *mepR* and *mepA*. MepR does not bind to the *mepB* promoter region [88]. The binding of MepR is stronger with the *mepA* promoter than with the *mepR* promoter, and the stoichiometry of this binding is probably different [89]. The *mepA* promoter presents both high- and low-affinity MepR binding sites that overlap with the -35 and -10 consensus sequences, resulting in a tight repression of *mepA *by MepR, which may bind as two dimers. On the other hand, the weaker and smaller MepR binding site in *mepR* promoter encompasses the transcription initiation site, located immediately downstream of the -10 consensus sequence, which can result in a weaker interaction with MepR that is thought to bind as a single dimer [89]. The weaker repression of *mepR* could explain the observation that only *mepR* transcripts are detected in wild-type strains [37,88]. Analysis of mutant strains overexpressing the *mepA* gene were found to have mutations in MepR, such as the introduction of premature stop codons and consequently the production of truncated forms of MepR that can render the protein non-functional, thus enabling the transcription of the *mepA* gene [37,89].

The auto-regulatory MepR is also responsive to the presence of MepA substrates, although the substrate specificity of MepA and MepR do not entirely overlap [88,90]. There is evidence that MepR binds to several MepA substrates, and it is postulated that the binding of the MepR substrate induces a change in MepR conformation so that the interaction between MepR and the DNA binding sites is diminished [89,90].

QacR is encoded by the gene *qacR* that occurs immediately upstream of the genes *qacA* and *qacB* being transcribed divergently to these genes [45]. QacR possesses a helix-turn-helix DNA binding motif, common to regulatory proteins, and belongs to the TetR family of transcriptional repressors [45]. It has been shown that QacR is a direct repressor of the expression of the *qacA *gene, by binding to the *qacA* promoter [91]. QacR binds to a large inverted repeat (IR1) that is located immediately downstream of the *qacA/B* promoters and overlaps the *qacA/B* transcription initiation sites [92]. The binding mechanism of QacR to IR1 differs from that of other TetR family of regulatory proteins, with two dimers of QacR binding cooperatively to IR1, not through protein-protein interactions but *via* a widened conformation of the DNA promoted by the binding of the first QacR dimer that favors the binding of the second one [93]. When bound to the IR1 in the *qacA* promoter, QacR represses the transcription of this gene [91]. However, upon addition of QacA substrates, *qacA* expression increases in a substrate concentration-dependent manner by inhibition of the QacR binding to the *qacA* promoter [91]. This inhibition is achieved by the direct interaction of QacR to the wide array of QacA substrates [91]. Thus, QacR is also a multidrug-binding protein, for which several structures of complexes QacR:drug have been determined and have revealed the presence of several, distinct, but linked binding sites within one extended and multifaceted binding pocket that has evolved to accommodate the broadest range of noxious hydrophobic molecules [94,95]. The binding of a QacA substrate to QacR results in a change in QacR conformation, which renders the protein unable to bind IR1, thus allowing *qacA* transcription [94]. Not all compounds that are QacA substrates were shown to induce *qacA* expression or to inhibit the QacR binding to IR1, suggesting that there is a basal *qacA* expression and thus QacR is a relatively weak repressor [91].

The increased activity or synthesis of the efflux pumps described above, which can occur in response to several chemical or environmental stimuli, potentiates the survival of antimicrobial resistant *S. aureus*, which may have relevant implications in the management of these infections in the clinical settings. 

## 5. CLINICAL SIGNIFICANCE OF MDR EFFLUX PUMPS IN *S. aureus*

In bacteria, clinically significant resistance mediated by drug efflux can encompass either antibiotics, biocides, or both. Efflux-mediated antibiotic resistance may involve efflux systems capable of extruding a single class of antibiotics, such as the case of the Tet determinants that convey resistance to tetracycline, or the extrusion of multiple classes of antibiotics by MDR efflux pumps, such as the ones described in this review. The overexpression of MDR efflux pumps, due to their promiscuous substrate specificity, can promote resistance to several classes of antibiotics but also decreased susceptibility to biocides, resulting into a multidrug resistance phenotype [7]. Besides the risk of therapeutic failure, the development of these multidrug resistance phenotypes may give rise to other problems, such as the possible co-selection and cross-resistance between efflux mediated antibiotic and biocide resistance, particularly worrisome for drug resistant strains such as MRSA strains.

## 5.1. Assessing Efflux Activity

To fully ascertain the role played by a given efflux system in antibiotic and/or biocide resistance, one must assure that the methodology applied is the most adequate to disclose efflux activity. The majority of the studies on efflux-mediated resistance among *S. aureus* clinical isolates use the decrease of antibiotic minimum inhibitory concentration (MIC) in the presence of compounds described to inhibit efflux activity, the so called efflux inhibitors (EIs) [27,96-1]. This approach is laborious and dependent on the susceptibility of the efflux system(s) to that particular inhibitor, which can vary considerably and for which the mechanism of action at the cellular level remains, in most cases, to be clarified [101-103]. 

More recently, ethidium bromide, a broad range efflux pump substrate, has been explored to assess efflux activity in *S. aureus* cells. This molecule can be used as a marker for the indirect assessment of efflux activity, namely, by determination of ethidium bromide MICs to identify *S. aureus* strains that show increased efflux activity [84,104] in the presence/absence of efflux inhibitors [22,105], by the Ethidium Bromide-Agar Cartwheel Method, which evaluates the cells capacity to retain/extrude ethidium bromide after overnight incubation [106] or in assays that evaluate directly the efflux activity, using real-time fluorometry [107].

## 5.2. Efflux-mediated Fluoroquinolone Resistance

Reports of antibiotic resistance mediated by efflux pumps in *S. aureus* include many classes of antibiotics, with particular emphasis for tetracyclines, macrolides and fluoroquinolones. Nevertheless, is on fluoroquinolones that much attention has been focused since this class of antibiotics includes many substrates of a large number of MDR efflux systems [108]. Fluoroquinolone resistance in *S. aureus* has been mainly attributed to mutations occurring in the cellular targets GrlA/GrlB (topoisomerase IV, encoded by genes *grlA/grlB*) and GyrA/GyrB (DNA gyrase, encoded by genes *gyrA/ gyrB*); which decrease their affinity to the drug, conferring high-level fluoroquinolone resistance [109-111]. Efflux-mediated resistance to fluoroquinolones, such as ciprofloxacin, norfloxacin and sparfloxacin, has been described in *S. aureus* clinical isolates for the last two decades, albeit sporadically [96-98,112-116]. However, the role played by each individual efflux system is difficult to establish, since the majority of the chromosomal MDR efflux pumps extrude fluoroquinolones. All these factors contribute for the relatively poor characterization of efflux-mediated fluoroquinolone resistance in clinical *S. aureus* isolates [117]. 

Very few reports relate the actual contribution of each chromosomal MDR efflux pump to the resistance of *S. aureus* towards fluoroquinolones. A study by DeMarco *et al*. reported the occurrence of 49% of strains showing increased efflux activity within a collection of 232 bloodstream *S. aureus* isolates [27]. The increased efflux activity present in those isolates was correlated with increased resistance to fluoroquinolones, biocides and dyes. Efflux pump gene expression assays revealed that among strains with increased efflux activity, nearly half (48%) overexpressed MDR efflux pumps. Of these last ones, 57% strains showed overexpression of a single efflux pump gene, most commonly *norA* or *mepA*, while the remaining 43% isolates overexpressed two or more efflux pump genes, predominantly *norB/C* [27]. A recent study from our group on a collection of 52 ciprofloxacin resistant isolates detected increased efflux activity in 23% of the isolates, which was associated with increased resistance to fluoroquinolones [105]. These isolates with increased efflux activity showed a strong involvement of efflux in the resistance phenotype, but assessment of gene expression in representative isolates revealed low levels of efflux pump gene expression, which occurred only in the presence of agents, such as ciprofloxacin. The opposite effect was observed for a pan-susceptible reference strain, suggesting that the clinical isolates could be already primed to respond to noxious compounds. In fact, these isolates are under a constant pressure by antimicrobial compounds, such as antibiotics and biocides. Since the expression of efflux pumps provides the cell with the means to cope with these compounds, it could be expected that those clinical isolates already have in their cell membrane the necessary number of efflux pump proteins [105]. We also observed that not only different substrates can trigger expression of different efflux pump genes for the same strain, but also that the same substrate can promote a variable response, according to its concentration. The pattern of gene expression encountered in this study showed some differences from the one found by DeMarco *et al.*, the most striking being the absence of *norA* overexpression [105]. A different MDR pumps expression pattern was also reported for *S. aureus* prototype strains [84]. Altogether, these studies reveal that *S. aureus* clinical isolates may diverge in the efflux-mediated response to noxious agents, including fluoroquinolones, even when highly clonally related [105].

## 5.3. Efflux-mediated Biocide Resistance

Biocides play an important role in infection control and hygiene, being extensively used in health care settings, consumer products, animal husbandry and food industry [118]. Resistance to biocides has been reported since the 1950s [119], with particular focus on bacterial resistance towards quaternary ammonium compounds used as disinfectants [120-122]. The action of biocides differs from the one of antibiotics by the multiplicity of their targets within the bacterial cell. Thus, emergence of resistance towards these compounds rarely occurs by mechanisms such as the modification of target sites. Instead, resistance to biocides is more often associated with the reduction of the biocide intracellular concentration, either by a decrease in cell wall permeability or efflux of the biocide [118]. In *S. aureus,* resistance to antiseptics and disinfectants has been mainly attributed to the plasmid-encoded efflux systems, QacA/B, Smr, QacG, QacH and QacJ. The few reports on the prevalence of these genes suggest that their distribution may follow a geographic variation, albeit more and larger studies are necessary to draw a more accurate portrait of this distribution. Studies with clinical isolates conducted in Asian countries have reported prevalences from 0% to 73% for *qacA/B* genes and 0 to 32% for the *smr* gene [123,124], although several reports from Japan spanning several years apart have reported a constant prevalence of around 35% and 3% for genes *qacA/B* and *smr*, respectively [55,125,126]. Reports from Europe have also described different prevalence values; Mayer *et al*. described a prevalence of 42% of *qacA/B* and 6% of *smr* genes among a collection of 497 clinical isolates [127], while others have reported values of 8.3% and 44% [128], and 15% and 4%, respectively [129]. In our own study of a collection of 53 *S. aureus* clinical isolates from a Portuguese hospital, we found 1.9% occurrence of *qacA* and *smr* genes [105, unpublished data]. A recent study with *S. aureus* clinical isolates from Tunisia reported the occurrence of 13% isolates carrying *qacA*, 9% carrying *qacB* and 11% with *smr* genes [130]. Another recent report from Canada revealed the occurrence of the *qacA/B* and* smr* genes in 2% and 7%, respectively, amongst a collection of 344 *S. aureus* clinical isolates [131]. The majority of these studies describe the co-occurrence of both *qacA/B* and* smr* genes in the same isolate [55,123,127-130].

The occurrence of other *qac* genes, namely *qacG*, *qacH* and *qacJ*, has been mainly described in isolates of animal origin and food industry [65,132]. However, a study reported the carriage of *qacH* gene by 3% of 120 MRSA isolates from the United Kingdom [128], and another recent study revealed the presence of these genes in *S. aureus* clinical isolates, but with very low-prevalence rates, 0.5% for *qacH*, 2.2% for *qacG* and 1.3% for *qacJ* [133]. This last study also referred the joint co-occurrence of *qacG* and *qacA/B* or *smr* [133].

The occurrence of the *qacA/B* gene is mainly associated with high-level resistance to several antiseptics, disinfectants and dyes. We have previously demonstrated the carriage of the *qacA* gene in a *ca*. 30-kb plasmid by the MRSA strain HPV107, representative of the so-called MRSA Iberian clone that is disseminated throughout several European countries and the USA [134]. By curing HPV107 of this plasmid and comparing the initial and the plasmid-cured culture, we could establish an association between carriage of this plasmid/QacA determinant and a higher efflux activity in strain HPV107, showing also that this activity was linked to the reduced susceptibility towards several biocide compounds showed by HPV107. In particular, the plasmid-cured derivative of HPV107 became more susceptible to all biocides tested, which included tetraphenylphosphonium bromide, dequalinium chloride, benzalkonium chloride, hexadecyltrimethylammonium bromide, cetrimide, and pentamidine, with exception of chlorhexidine, for which no alteration was detected, and to the dyes ethidium bromide, berberine, acriflavine and crystal violet. On the other hand, no alterations were detected in the antibiotic-resistant profile of the plasmid-cured strain [134]. 

Chromosomally encoded MDR efflux pumps are also capable of conferring resistance to biocides. In *S. aureus*, all MDR efflux pumps encoded in the chromosome have the potential to extrude biocides and are associated with low- to high-level resistance to these compounds. However, the assessment of their involvement in biocide resistance is more difficult to ascertain, since these efllux pumps occur naturally in *S. aureus*. Several studies have associated a biocide resistance phenotype in clinical isolates with the overexpression of efflux pump genes. Huet *et al*. demonstrated that the selective pressure of biocides could result in increased resistance to these compounds due to the overexpression of efflux pumps such as *mepA*, *norA*, *norC* and *mdeA* [28]. 

## 5.4. The role of Efflux on the Relation between Antibiotic and Biocide Resistance

The question of whether biocide resistance can have a role on the selection of antibiotic-resistant strains has been a matter of debate [118,120,135,136]. Data available on literature report contradictory data concerning the relation between antibiotic and biocide resistance, but evidence has been gathered supporting the occurrence of co-selection of strains with reduced susceptibility to biocides by antibiotic-resistant bacteria, and *vice-versa* [137-141]. 

In *S. aureus*, many MDR efflux pumps involved in biocide resistance are encoded on large conjugative plasmids that can also carry other resistance determinants [52]. One such example is the linkage between *qacA* and the gene *blaZ*, which encodes a β-lactamase. Several reports have described an association between biocide resistance and penicillin resistant staphylococci isolates of animal [142] and human origin [143], due to the dissemination of multiresistance plasmids carrying both *qacA/B* and *blaZ*. The *qacA* gene can also be encountered in plasmids together with genes encoding resistance to trimethoprim, aminoglycosides, fosfomycin and heavy-metals [144-146].

Another point of debate is the co-selection of MRSA strains by strains harbouring *qac* genes. Although some studies have found no linkage between MRSA and *qacA/B* carriage [147], others studies suggest the opposite [55, 127]. A recent study by Zhang *et al*. has showed that *qac* genes were more frequent in MRSA strains that colonized nurses than the general public [148].

The role played by efflux pumps on the development of resistance to several types of antimicrobial agents has been demonstrated by the step-wise adaptation of a susceptible strain to ethidium bromide, which resulted in an adapted strain with increased resistance not only to ethidium bromide but to several other antimicrobial compounds, including fluoroquinolones. This multidrug resistance phenotype was due to the overexpression of a single MDR efflux pump, NorA [22]. Huet *et al*. have also shown that single- and multi-step exposure of *S. aureus* strains to different biocides and dyes resulted in MDR phenotypes, by overexpression of efflux pump genes [28].

## 6. CONCLUSIONS

For a long time, the role of efflux pumps in antibiotic resistance was neglected, in particular for Gram-positive bacteria. The last decade witnessed a significant build-up of information on these systems, in particular on MDR efflux pumps. Nevertheless, our current understanding on how these systems operate and respond to the environmental pressure created by antimicrobial agents is still relatively dispersed. 

Several aspects have prevented the understanding of a clear picture of the real role efflux pumps play in the appearance of bacterial resistance to antimicrobial compounds.

First, the fact that the level of resistance attributable to efflux-mediated resistance is, in most of the cases, lower than the one conferred by the other resistance mechanisms and sometimes difficult to establish, depending on the method used to evaluate efflux activity. Second, bacterial response to these compounds is mediated, in most of the cases, not by a single but by several efflux pumps, that react in a concerted manner to the presence of the antimicrobial, which difficults the perception of the role played by each individual pump on the overall efflux activity/resistance phenotype. Third, bacterial efflux systems are regulated by several specific and/or global regulators that act in an intricate network of regulatory/sensory pathways, which may confuse our interpretation of data. Finally, there is still a lack of consensual methodological approaches that guarantee appropriate address of all these issues.

The pertinence of these questions becomes more obvious when studying efflux activity in collections of clinical strains, for which the myriad of responses difficult even more a clear interpretation of data. The few studies of efflux-mediated antimicrobial resistance on *S. aureus* of clinical origin revealed that more than a specific efflux-mediated response to a specific stimuli (antimicrobial agent), one may observe distinct responses to the same stimuli. This probably results from the specific effect of that stimulus on each specific strain, as the result of the strain genetic background and responsive efflux systems. 

It is established that efflux pumps contribute to low level resistance to antimicrobial agents and the emergence of MDR phenotypes in clinical strains by the extrusion of multiple, unrelated compounds, including antibiotics and biocides. The interrelations between these two groups of anti-microbials in the inducement of resistance is not yet clarified but the promiscuous activity of MDR efflux pumps foresee a close relation in the development of efflux-mediated resistance to both types of antimicrobials.

Recent studies from independent groups, working with different bacteria, suggest that efflux systems may be a first response to the antimicrobial making possible for the cell to survive and acquire other, more stable resistance mechanisms that will then provide a high-level resistance phenotype, as was recently demonstrated for *E. coli* [149] and *Mycobacterium tuberculosis* [150]. In *S. aureus*, some evidences have been also found for this role of efflux pumps as a first-line defense mechanism towards noxious compounds [151,152] that are supported by data on clinical strains [27,28,105].

All these observations open new perspectives for the future work on efflux pumps, which may include a new therapeutic concept on the usefulness of efflux inhibitors as adjuvants of the conventional anti-bacterial therapy to overcome the scarcity of therapeutic approaches for these MDR infections [100]. 

## Figures and Tables

**Table 1.** Multidrug Efflux Pumps Described for *Staphylococcus aureus*

Efflux Pump	Family^a^	Regulator(s)^b^	Substrate Specificity	References
Chromosomally-encoded Efflux Systems				
NorA	MFS	MgrA, NorG(?)	Hydrophilic fluoroquinolones (ciprofloxacin, norfloxacin) QACs (tetraphenylphosphonium, benzalkonium chloride) Dyes (e.g. ethidium bromide, rhodamine)	[16,18,19]
NorB	MFS	MgrA, NorG	Fluoroquinolones (e.g. hydrophilic: ciprofloxacin, norfloxacin and hydrophobic: moxifloxacin, sparfloxacin) Tetracycline QACs (e.g. tetraphenylphosphonium, cetrimide) Dyes (e.g. ethidium bromide)	[31]
NorC	MFS	MgrA(?), NorG	Fluoroquinolones (e.g. hydrophilic: ciprofloxacin and hydrophobic: moxifloxacin) Dyes (e.g. rhodamine)	[35,36]
MepA	MATE	MepR	Fluoroquinolones (e.g. hydrophilic: ciprofloxacin, norfloxacin and hydrophobic: moxifloxacin, sparfloxacin) Glycylcyclines (e.g. tigecycline) QACs (e.g. tetraphenylphosphonium, cetrimide, benzalkonium chloride) Dyes (e.g. ethidium bromide)	[37,38]
MdeA	MFS	n.i.	Hydrophilic fluoroquinolones (e.g. ciprofloxacin, norfloxacin) Virginiamycin, novobiocin, mupirocin, fusidic acid QACs (e.g. tetraphenylphosphonium, benzalkonium chloride, dequalinium) Dyes (e.g. ethidium bromide)	[39,40]
SepA	n.d.	n.i.	QACs (e.g. benzalkonium chloride) Biguanidines (e.g. chlorhexidine) Dyes (e.g. acriflavine)	[41]
SdrM	MFS	n.i.	Hydrophilic fluoroquinolones (e.g. norfloxacin) Dyes (e.g. ethidium bromide, acriflavine)	[42]
LmrS	MFS	n.i.	Oxazolidinone (linezolid) Phenicols (e.g. choramphenicol, florfenicol) Trimethoprim, erythromycin, kanamycin, fusidic acid QACs (e.g. tetraphenylphosphonium) Detergents (e.g. sodium docecyl sulphate) Dyes (e.g. ethidium bromide)	[43]
*Plasmid-encoded Efflux Systems*				
QacA	MFS	QacR	QACs (e.g. tetraphenylphosphonium, benzalkonium chloride, dequalinium) Biguanidines (e.g. chlorhexidine) Diamidines (e.g. pentamidine) Dyes (e.g. ethidium bromide, rhodamine, acriflavine)	[45,49]
QacB	MFS	QacR	QACs (e.g. tetraphenylphosphonium, benzalkonium chloride) Dyes (e.g. ethidium bromide, rhodamine, acriflavine)	[53]
Smr	SMR	n.i.	QACs (e.g. benzalkonium chloride, cetrimide) Dyes (e.g. ethidium bromide)	[58,61]
QacG	SMR	n.i.	QACs (e.g. benzalkonium chloride, cetyltrymethylammonium) Dyes (e.g. ethidium bromide)	[67]
QacH	SMR	n.i.	QACs (e.g. benzalkonium chloride, cetyltrymethylammonium) Dyes (e.g. ethidium bromide)	[68]
QacJ	SMR	n.i.	QACs (e.g. benzalkonium chloride, cetyltrymethylammonium) Dyes (e.g. ethidium bromide)	[69]

a n.d.: The family of transporters to which SepA belongs is not elucidated to date.

b n.i.: The transporter has no regulator identified to date.

QACs: quaternary ammonium compounds
